# Validity of a Mobile Application to Diagnose Temporomandibular Disorders

**DOI:** 10.3390/jcm12227193

**Published:** 2023-11-20

**Authors:** Yoo-Ree Hong, Na-Kyung Hwangbo, Alec-Hyung Kim, Seong-Taek Kim

**Affiliations:** 1 Department of Orofacial Pain and Oral Medicine, Yonsei University College of Dentistry, Yonsei-ro 50-1, Seodaemun-gu, Seoul 03722, Republic of Korea; yrhong0525@gmail.com (Y.-R.H.); 1014hwangbo@gmail.com (N.-K.H.); 2 TMJ & Orofacial Pain Clinic, Los Angeles, CA 90006, USA; sontec-kim@daum.net

**Keywords:** temporomandibular joint, diagnostic application, digital health, digital therapeutics

## Abstract

This study aimed to assess the diagnostic accuracy of a mobile application by comparing its diagnoses to those of Orofacial Pain and Oral Medicine specialists and further imaging results (CBCT and MRI) in 500 patients with temporomandibular disorder (TMD). The research focused on three diagnostic categories: the initial specialist diagnoses, the final diagnoses after imaging, and the mobile app’s diagnoses. The concordance rates, sensitivities, specificities, and positive predictive values of the diagnoses were examined, with further imaging serving as the gold standard. The mobile app demonstrated a high concordance rate compared to both the final (0.93) and the initial specialists’ diagnoses (0.86). The sensitivities, specificities, and positive predictive values also indicated strong reliability, affirming the app’s diagnostic validity. Although the concordance rate was slightly lower when comparing the app’s diagnoses to the imaging results (CBCT and MRI), the specialists’ diagnoses yielded similar results. The study suggests that user-friendly diagnostic mobile applications, based on the diagnostic criteria for TMD, could enhance the clinical management of TMD. Given the reliability of mobile applications for diagnostic purposes, their wider implementation could facilitate the provision of appropriate and timely treatments for patients with TMD.

## 1. Introduction

Recently, dentistry has focused on the explosive growth of digital health worldwide, which encompasses concepts such as social media platforms, mobile applications, wearable devices, cloud-based data platforms, and telemedicine. Digital health has expanded the scope of general well-being and health monitoring from limited activities in hospitals and clinics to a vast digital world through various applications [1,2,3]. Research on innovative models has been undertaken in the digitized health domain, utilizing open-source engineering and optimizing the potential of experts in traditional medical environments. Digital Therapeutics (DTx) is a subset of digital health that provides evidence-based therapeutic interventions driven by software to prevent, manage, or treat medical disorders in patients [1,4]. Software-based DTx options are gaining attention because they extend beyond the physical boundaries of traditional clinics or hospitals, aiming to bridge the gap in healthcare delivery [1].

Temporomandibular disorder (TMD) is a condition characterized by functional impairment of the temporomandibular joint (TMJ) and the musculoskeletal system of the head and neck [5]. It is a common condition that causes orofacial pain and affects approximately 10–15% of the population [6]. Moreover, it is considered a growing condition in modern society, with reported prevalence rates, where at least one symptom is present, of 40–75% among adults in the United States. Symptoms include localized pain in the preauricular area and/or masticatory muscles, jaw motion abnormalities, and articular sounds during mandibular movement. The etiology of TMD is multifactorial and involves neuromuscular, neurobiological, biomechanical, and biopsychosocial factors [5,6,7]. Certain parafunctional habits, such as clenching and bruxism, are potential risk factors [8]. The incidence of TMD has been increasing due to factors such as stress, lifestyle habits, and tooth grinding (bruxism). It has become a prevalent condition in the modern age, showing a prevalence increase of over 10% compared to previous years; this has been primarily influenced by psychological factors, especially after the COVID-19 pandemic [9,10].

TMD is a complex disorder that requires a comprehensive diagnosis by oral and maxillofacial specialists, owing to its multifactorial causes and diverse symptoms. It is important to differentiate muscle pain from joint problems, which often necessitate the interpretation of imaging studies and specialized knowledge. Additionally, because TMD tends to progress chronically or has a high likelihood of progression, it is crucial for specialists to establish an appropriate treatment plan [11,12]. The diagnosis of TMD is complex, and imaging instruments do not clearly detect the main symptoms. The research diagnostic criteria (RDC/TMD) and diagnostic criteria (DC/TMD) aim to standardize TMD diagnosis for research and clinical purposes [5,11].

The increasing number of patients with TMD influenced by psychological stress poses challenges due to a shortage of specialists in orofacial pain and oral medicine. More than 68% of World Health Organization (WHO) member countries report having fewer than five dentists per 10,000 people (approximately 37% report having fewer than one dentist). In most countries, dental care systems are insufficient to meet the demands of oral health management. Additionally, the proportion of countries implementing specialized dental practitioner systems is low, and the availability of specialists in oral medicine is either nonexistent or very limited. According to the 2019 NLO Report Compilation, as of 2020, there were 31,633 dentists in South Korea, which ranked 13th worldwide. However, there were only 217 specialists in oral medicine among them, accounting for 0.69% of the total, which is a very low proportion. In a relatively recent development, in 2020, the National Commission on Recognition of Dental Specialties and Certifying Boards (NCRDSCB), an ADA subsidiary, recognized oral medicine as the eleventh dental specialty. This imbalance hinders timely treatment of TMD patients. 

In clinical settings, the DC/TMD diagnostic process can be challenging for non-specialists because of its unfriendly user interface, multiple steps, and time-consuming nature. To address this issue, one study developed a diagnostic tool in a checklist format [13], and many other studies tried to follow the DC/TMD format in a simpler manner [14,15] to facilitate a quick and accurate TMD diagnosis by increasing the number of clinicians who could manage TMD. However, the reality was that general practitioners faced limitations in making accurate diagnoses and treatment plans based solely on patients’ symptoms, without specialized imaging equipment such as CBCT or MRI. This has led to a growing recognition of the need for DTx to assist general practitioners in clinical settings by enabling a relatively straightforward diagnosis and treatment planning. In our study, we utilized a mobile application that follows DC/TMD decision trees to diagnose patients with TMD and to provide a simple treatment plan. This application was developed as a program to assist general clinicians in accurately diagnosing patients and establishing treatment plans, as well as facilitating insurance claims by inputting patients’ symptoms. By promoting wider usage of the DC/TMD tool, the correct diagnosis and management of TMD can become more common. However, studies on the development, implementation, and evaluation of such applications in dentistry and TMD are lacking.

Therefore, the primary objective of this study was to evaluate the diagnostic validity of the diagnostic mobile application compared to specialists’ diagnoses. The secondary objective was to evaluate the concordance rates between the results of CBCT and MRI, which are considered the gold standard of imaging techniques for TMD, and the diagnoses by both the application and the experts [16]. The null hypothesis states that the application lacks diagnostic validity compared to the experts’ diagnoses.

## 2. Materials and Methods

This study evaluated three different diagnoses: the specialists’ provisional diagnoses, their final diagnoses, and diagnoses from the application. The concordance rates, sensitivities, specificities, and PPVs of these diagnoses were assessed. In addition, the concordance rates of the diagnoses were compared with the results of further imaging, which served as the gold standard. A comprehensive evaluation was performed considering the clinical manifestations of the joint sounds and their correlation with the diagnoses:

(1) Comparing the concordance rates between the tool and specialists’ diagnoses;
(2) Calculating sensitivities, specificities, and PPVs for seven different diagnostic impressions;
(3) Conducting additional evaluation using further imaging;
(4) Comparing the clinical diagnoses made by specially trained clinicians with the actual imaging results.

### 2.1. Subjects

This study was approved by the Institutional Review Board of the Dental Hospital (IRB number: 2-2021-0006). A total of 500 patients aged 12–78 years (mean age, 37.4; 77.4% women) were randomly selected from the Department of Orofacial Pain and Oral Medicine at Yonsei University Dental Hospital in Seoul, Korea. The study period ranged from 1 January 2021 to 31 December 2021. The study included patients who presented with TMD and orofacial pain and who were diagnosed with conditions such as muscle pain, spasm, TMJ arthralgia, degenerative joint disease (DJD), disc displacement, condyle luxation, or bruxism. Patient charts were reviewed to collect information on sex, age, joint sounds, occlusion, parafunctional habits, range of jaw motion, pain intensity, and pain location. Patients already undergoing other treatments for TMD (such as medications or oral appliances) were excluded from the study.

Among the 500 patients, 100 underwent TMJ CBCT, and 48 underwent TMJ MRI. These patients were selected for further evaluation to assess the diagnostic validity of the mobile application.

### 2.2. Methods

#### 2.2.1. Specialists’ Diagnoses

The specialists were three dental specialists who had diagnosed patients in the Department of Oral Medicine of a dental hospital for more than 15 years. The patients underwent a comprehensive assessment that included a clinical examination and three plain radiographs: a panoramic view, TMJ panoramic view, and skull P-A view. Clinical examinations involved palpation of the TMJ and masticatory muscles, measurement of jaw motion ranges (maximum mouth opening, protrusion, and lateral movement), and evaluation of TMJ sounds using an orofacial pain chart with a numeric rating scale (NRS) ranging from 0 to 10. Additional evaluations included assessment of cheek ridging, tongue ridging, and attrition. Diagnoses were made according to the diagnostic criteria for TMD. Specially trained examiners performed clinical examinations and made the diagnoses. If necessary, further imaging, such as TMJ MRI and/or TMJ CBCT, was performed after the initial visit. In this study, “provisional diagnoses” refers to the specialists’ provisional diagnoses based solely on the plain X-rays, while “final diagnoses” refers to the diagnoses made after additional imaging.

#### 2.2.2. Diagnoses by the Diagnostic Mobile Application

**Figure 1:**
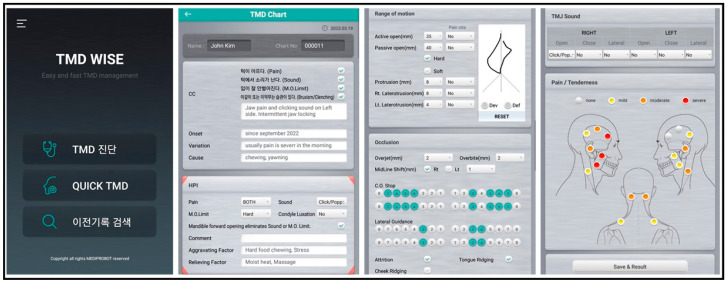
Screenshots showing representative content of the diagnostic mobile application for TMD.

The mobile application’s (TMD WISE^®^, Mediprobot Co., Seoul, Republic of Korea) diagnoses were made using simple questionnaires in which the examiner clicked a dot and provided a description of the present illness based on the orofacial pain evaluation chart. The questionnaire covered chief complaints, pain location, pain severity (mild, moderate, severe), habits, TMJ sounds (click, crepitus), jaw range of motion, and occlusion. Based on the information entered into the diagnostic tool, an impression, treatment plan, and subsequent plans were provided. It is important to note that these diagnoses did not include the reading of plain X-rays. Treatment plans included 666 exercises, heat packs, rest, NSAID and muscle relaxant prescriptions, and splints (Figure 1). The same specialists who made the diagnoses used the application, and their diagnosis results were compared with the diagnoses provided by the application. 

#### 2.2.3. Further Imaging

Patients with suspected bony changes related to DJD or disc displacement underwent additional imaging using TMJ CBCT and/or TMJ MRI. TMJ CBCT was performed using the Rayscan Symphony machine (RAY Co., Ltd., Hwaseong, Republic of Korea). TMJ MRI scans were conducted using a 3.0-T unit (Pioneer; GE Healthcare, Waukesha, WI, USA) and included multiplanar spin-echo T1-weighted imaging (TR/TE, 734/9) and multiplanar spin-echo T2-weighted imaging (TR/TE, 2707/66). These imaging procedures were performed at the Department of Oral and Maxillofacial Radiology, Yonsei University Dental Hospital, within two weeks of the patient’s initial visit.

Comprehensively, three diagnoses were compared: the specialists’ provisional diagnoses based on plain radiographs, the final diagnoses after further imaging, and diagnoses of the diagnostic mobile application.

### 2.3. Statistical Analysis

The sensitivities, specificities, and positive predictive values (PPVs) of the application’s diagnoses were assessed and compared with those of the specialists. The concordance rate between the two diagnoses made by the specialists and the tool, as well as the concordance rate with further imaging, was also evaluated. These values were calculated using a method similar to those reported in previous studies [17].

## 3. Results

### 3.1. Validity Assessment

The concordance rates of the diagnoses by the application against the specialists’ final diagnoses and provisional diagnoses were 0.92921 and 0.855718, respectively (Table 1). The sensitivities, specificities, and PPVs are summarized in Table 2, with the sensitivities ranging from 0.75 to 1.0, the specificities ranging from 0.925403 to 0.969499, and various PPVs ranging from 0.075 to 1.0. “Disc displacement with reduction” included disc displacement with reduction and intermittent locking, whereas “disc displacement without reduction” included disc displacement without reduction with and without limited opening. Myalgia included both local and myofascial pain.

### 3.2. Imaging

This study compared the concordance rates of the CBCT and MRI results with the diagnoses made by the specialists and the application. The concordance rate for CBCT and the specialists’ diagnoses was 0.73232, whereas the concordance rate for MRI and the specialists’ diagnoses was 0.645833. The concordance rate for CBCT and the application’s diagnoses was 0.6122, and the concordance rate for MRI and the application’s diagnoses was 0.58333 (Table 3 and Table 4).

## 4. Discussion

This study aimed to assess the diagnostic validity of a diagnostic mobile application by comparing its diagnoses with specialists’ clinical diagnoses. These analyses were conducted to evaluate the accuracy and reliability of the mobile diagnostic application in diagnosing TMD, considering its concordance with the specialists’ diagnoses and its correlation with the imaging findings. The evaluation comparing the provisional diagnoses, the final diagnoses by specialists, and the diagnoses from CBCT, MRI, and the application revealed a high level of agreement between the application’s and the specialists’ final diagnoses (including CBCT or MRI). This observation suggests that the application is effective in diagnosing patients with TMD. Additionally, when comparing the application’s diagnoses with the imaging results (CBCT and MRI), a slightly lower concordance rate was observed. Therefore, the null hypothesis was rejected.

The diagnostic mobile application used in this study was developed to compensate for the low accessibility in clinical settings, owing to the low specialist-to-patient ratio, and aims to provide rapid diagnosis and treatment for patients with TMD, a condition that is rapidly increasing and prone to becoming chronic [18,19,20,21]. This study included 500 randomly selected participants with an average age of 37.4 years and a female-to-male ratio of 77.4%. This composition aligns with previous studies that reviewed the prevalence of TMD, which reported an average age range of 30–40 years and a sex ratio of 1:3.3, confirming the consistency of this study’s participant composition [22,23,24,25].

The concordance rates between the application’s diagnoses and the specialists’ final diagnoses and provisional diagnoses were high, with values of 0.92921 and 0.855718, respectively. The observed differences in the concordance rates between the diagnoses made by clinicians and the application can be attributed to several factors [26]. First, clinicians have the advantage of directly assessing plain radiographs, which can provide valuable information for diagnosing conditions such as severe DJD [27,28]. The application, on the other hand, does not have the capability to interpret X-rays, which may result in a lower concordance rate when considering provisional diagnoses. Furthermore, the application focused solely on the symptoms present during the clinical examination. It does not consider the patient’s history of chief complaints or symptoms, which can provide valuable information for differential diagnosis. Consequently, the lower concordance rate observed for this application could be attributed to the exclusion of historical information that clinicians may have considered when formulating their diagnoses [29,30].

Calculations of sensitivity, specificity, and PPV provide valuable insights into the reliability of the diagnostic tool [17,31,32,33]. While most impressions showed high values for these measures, three values were notably low: the PPV of disc displacement without reduction, the sensitivity of DJD, and the PPV of spasm. The low PPV of disc displacement without reduction can be attributed to the difficulty clinicians face in identifying this condition, as disc displacement without reduction without limited opening does not present with abnormalities in the mandibular range of motion or pain in clinical practice [26,34].

The low sensitivity of DJD can be explained by the fact that crepitus sounds are key indicators of this condition [35,36]. The absence of crepitus during clinical examination can lead to missed diagnoses, resulting in a low sensitivity. The remarkably low PPV of spasm is influenced by fully packed appointments at orofacial pain and oral medicine clinics. Because patients with acute symptoms often visit after their symptoms have subsided, owing to the long waiting period, the true positives of spasms are reduced, resulting in a low PPV.

Overall, these findings highlight the dependence of the diagnosis on the specific moment of clinical examination, emphasizing the importance of considering the limitations and context of the examination when interpreting the results. For instance, a patient who previously experienced jaw locking, pain in the left TMJ, and crepitus in the right TMJ but did not exhibit any abnormalities during the current examination could be diagnosed by a clinician as having disc displacement without a reduction in the left TMJ. However, a diagnostic tool that focuses solely on the current condition may classify the condition as normal because no notable abnormalities are present. Similarly, if a patient who has a history of crepitus and radiographic imaging shows suspicious changes in the bone structure, a clinician might suspect DJD of the right TMJ. Nevertheless, diagnostic tools may not consider these historical factors or radiographic evidence and categorize the condition as normal. These examples emphasize the limitations of relying solely on findings from a single clinical examination, highlighting the importance of considering a patient’s complete medical history and utilizing other diagnostic information for a comprehensive and accurate diagnosis.

The accuracy of the diagnoses was verified by further imaging using CBCT and/or MRI. CBCT was used to assess actual bony changes related to DJD, while TMJ MRI was used to evaluate disc displacement and other potential organic conditions. The concordance rates between the diagnoses made using the application and the actual imaging results were lower for both CBCT and MRI. In the case of MRI, this lower concordance rate can be attributed to the limitations of its application in capturing past medical history and intermittent symptoms as well as its inability to identify significant bony changes that may be visible on plain radiographs in cases of DJD [16,37]. Notably, there was little difference in the concordance rate between the diagnoses made by specially trained clinicians and those made by the application. This suggests that the diagnostic accuracy of the application was comparable to that of the experienced clinicians in this study.

The findings indicated that the application showed a lower concordance rate with actual imaging results, particularly in cases involving past medical history, intermittent symptoms, and prominent bony changes visible on plain radiographs. The application showed a similar concordance rate with the diagnoses made by specially trained clinicians. However, research findings and previous studies on DTx [38] cannot replace diagnosis and treatment by specialists. In summary, this application is useful as a diagnostic tool for TMD, particularly in areas with a shortage of dental specialists. Furthermore, it can serve as a supplementary tool for clinicians to diagnose TMD, offering an alternative to referring patients to higher-level healthcare institutions with limited availability. Therefore, its clinical use is recommended and encouraged. However, further research on the long-term application of such tools is necessary, as it is anticipated that DTx will contribute to resolving healthcare disparities in medically underserved areas [18].

One limitation of this study is its relatively small sample size, which limits the generalizability of the findings. Further long-term research with larger sample sizes is warranted. Additionally, the results of this study were evaluated based on specific parameters, which may be influenced by various factors, such as different diagnostic methods, settings (e.g., dental clinics instead of tertiary medical institutions), and evaluations by different examiners. Subsequent studies and additional investigations are required to further explore the effects of this application on more diverse parameters. A follow-up study should be conducted to confirm the application’s feasibility and validity by having general dentists use it in general dental clinics with an expected lower prevalence of temporomandibular disorders compared to the situation in a specialist clinic. Furthermore, future researchers should consider conducting subgroup analyses within the experimental group, such as distinguishing between bilateral TMJs and further categorizing specific diagnostic subgroups. Additional studies are necessary to comprehensively understand this topic. It is anticipated that the incorporation of various imaging modalities, such as TMJ panoramic radiography, CBCT, and MRI, into the application in the future will contribute to the advancement of DTx in the dental field. This method is expected to provide rapid and accurate diagnosis and treatment planning for patients with TMD.

## 5. Conclusions

In conclusion, this study assessed the diagnostic validity of the diagnostic mobile application by comparing its diagnoses to those of specialists and to CBCT and MRI results. Despite the limitations of our study, our findings suggest that the application offers accurate diagnostic results in dental clinical settings. While not as precise as an oral medicine specialist, it provides a precise and efficient tool for the diagnosis of TMD. When applied in routine clinical practice with the guidance of oral medicine specialists, it has the potential to streamline the diagnostic process and ensure the timely and accurate management of patients with TMD.

## Figures and Tables

**Table 1:** Concordance rates of diagnoses of the application against specialists’ diagnoses.

	Concordance Rate
Final diagnoses	0.92921
Provisional diagnoses	0.855718

**Table 2:** Sensitivities, specificities, and positive predictive values of the application’s diagnoses and numbers of diagnosed patients.

Impression	Sensitivity	Specificity	Positive Predictive Value	* Number of Diagnosed Patients
Arthralgia	0.891813	0.955696	0.977564	342
Disc displacement with reduction	0.950207	0.953668	0.950207	242
Disc displacement without reduction	0.97561	0.969499	0.740741	44
Degenerative joint disease	0.804348	0.949339	0.616667	50
Muscle pain	0.913525	0.938776	0.992771	454
Spasm	0.75	0.925403	0.075	10

* One patient may have received multiple diagnoses, leading to diagnostic duplication. Additionally, we did not differentiate between symptoms on the left and right sides of the body.

**Table 3:** Concordance rates of imaging (disc displacement with or without reduction).

	MRI
* Specialists’ diagnoses	0.645833
Application’s diagnoses	0.583333

* Specialists’ diagnoses: Diagnoses by specialists after reviewing electronic medical records and three general images.

**Table 4:** Concordance rates of imaging (degenerative joint disease).

	CBCT
* Specialists’ diagnosis	0.73232
Application diagnosis	0.6122449

* Specialists’ diagnoses: Diagnoses by specialists after reviewing electronic medical records and three general images.

## Data Availability

The data presented in this study are available on request from the corresponding author.
